# Plasmodesmata-Involved Battle Against Pathogens and Potential Strategies for Strengthening Hosts

**DOI:** 10.3389/fpls.2021.644870

**Published:** 2021-06-03

**Authors:** Jie Liu, Lin Zhang, Dawei Yan

**Affiliations:** ^1^State Key Laboratory of Crop Stress Adaptation and Improvement, School of Life Sciences, Henan University, Kaifeng, China; ^2^Joint International Research Laboratory of Agriculture and Agri-Product Safety of the Ministry of Education, Yangzhou University, Yangzhou, China

**Keywords:** plasmodesmata, plant pathogens, disease resistance, callose, callose synthase, cell-to-cell movement

## Abstract

Plasmodesmata (PD) are membrane-lined pores that connect adjacent cells to mediate symplastic communication in plants. These intercellular channels enable cell-to-cell trafficking of various molecules essential for plant development and stress responses, but they can also be utilized by pathogens to facilitate their infection of hosts. Some pathogens or their effectors are able to spread through the PD by modifying their permeability. Yet plants have developed various corresponding defense mechanisms, including the regulation of PD to impede the spread of invading pathogens. In this review, we aim to illuminate the various roles of PD in the interactions between pathogens and plants during the infection process. We summarize the pathogenic infections involving PD and how the PD could be modified by pathogens or hosts. Furthermore, we propose several hypothesized and promising strategies for enhancing the disease resistance of host plants by the appropriate modulation of callose deposition and plasmodesmal permeability based on current knowledge.

## Introduction

Throughout their life span, plants are constantly challenged by pathogens, namely fungi, bacteria, and viruses (Jones and Dangl, 2006). It is well-known that plant cells can respond to pathogens autonomously. Plants recognize microbe-associated molecular patterns (MAMPs) via pattern recognition receptors (PRRs) on the cell membrane, which initiates a series of signaling events and activates pattern-triggered immunity (PTI; Ranf, 2017; Saijo et al., 2018). Some pathogens, however, can produce effectors capable of inhibiting PTI to overcome the host immune system (Grant et al., 2006; Le Fevre et al., 2015; Toruno et al., 2016). A second defense response, which is activated by recognizing pathogenic effectors with corresponding nucleotide-binding leucine-rich repeat (NLR) proteins of hosts, is called effector-triggered immunity (ETI; Cui et al., 2015). Recently, the PTI and ETI systems were found to share common elements and to interact with each other (Ngou et al., 2021; Yuan et al., 2021). Besides the cell-autonomous immunity within infected regions, uninfected host cells could also establish immune responses in what is known as systemic acquired resistance (SAR; Klessig et al., 2018). To gain SAR, signaling molecules must move from infected cells to distal uninfected tissues (Wendehenne et al., 2014; Singh et al., 2017). Further, SAR confers an immune “memory” in hosts enabling them to activate defense responses more quickly and effectively when exposed to another pathogen attack (Conrath, 2006; Ramirez-Prado et al., 2018; Hake and Romeis, 2019; Guerra et al., 2020).

Plant cells have evolved unique cell-wall-spanning structures, termed PD, that link neighboring cells for their symplastic communication (Epel, 1994; Lucas et al., 2009). The typical PD are composed of plasma membrane (PM), cytoplasmic sleeve, and desmotubule derived from the endoplasmic reticulum (ER) (Zambryski and Crawford, 2000; Zambryski, 2008). Various key PD-localized proteins and lipids have been identified, including actin, receptor-like kinases, glycosylphosphatidylinositol-anchor proteins, remorins, sphingolipids, and sterols (Fernandez-Calvino et al., 2011). By controlling the intercellular exchange of both micromolecules and macromolecules, PD are functionally critical during the development of plants and in their responses to abiotic and biotic stresses (Maule, 2008; Lee and Lu, 2011; Lee et al., 2011; Han et al., 2014; Lee, 2014; Sager and Lee, 2014; Cui and Lee, 2016; Wu et al., 2016; Reagan et al., 2018; Miyashima et al., 2019; Yan and Liu, 2020). The aperture of the PD pore, which determines the size exclusion limit (SEL), is a major determinant of PD permeability (Lucas and Lee, 2004; Peters et al., 2021). This PD aperture is dynamically controlled by the deposition and degradation of callose within the cell walls near the neck of PD (Amsbury et al., 2017; Wu et al., 2018). Callose synthases (CalSs) and β-1,3 glucanases (BGs) govern the production and degradation of callose, respectively, fulfilling crucial roles in various developmental and physiological processes of plants (Chen and Kim, 2009; Zavaliev et al., 2011). PD-LOCALIZED PROTEINS (PDLPs) and PLASMODESMATA CALLOSE-BINDING PROTEINS (PDCBs) are two key protein families that positively regulate the dynamics of callose accumulation at PD (Simpson et al., 2009; Lee et al., 2011). Apart from callose, the architecture of PD also affects their conductivity and functioning. In this respect, PD may be classified as type I or II according to the status of the cytoplasmic sleeve between the PM and desmotubule. Compared with type II, the structure of type I PD lacks a visible cytoplasmic sleeve and internal tethers (Nicolas et al., 2017). Loss of function of the *PHLOEM UNLOADING MODULATOR* gene results in the lack of type II PD, whereas enhances PD permeability, fosters phloem unloading, and accelerates root elongation (Yan et al., 2019).

Beyond the key roles in plant development, the PD can participate in plant-pathogen interactions (Faulkner et al., 2013; Wang et al., 2013; Brunkard and Zambryski, 2017; Cheval and Faulkner, 2018). Specifically, PD facilitate the intercellular transport of mobile signal molecules, such as azelaic acid and glycerol-3-phosphate, needed for the establishment of SAR (Singh et al., 2017). Yet many pathogens and effectors can also spread in a cell-to-cell manner via PD to hasten the infection (Lent et al., 1991; Waigmann et al., 1994; Benitez-Alfonso et al., 2010; Cao et al., 2018). Currently, it remains an open question how plants modulate the timing of PD closure and the movement of SAR signals and pathogenic effectors to achieve the defense response. We speculate the apoplastic trafficking of immune molecules might provide an alternative way to impede the spread of pathogens or effectors in the case of blocked PD (Lim et al., 2016b; Singh et al., 2017). Further study of PD in the battle between plants and pathogens is gaining interest and becoming important. Here, we summarize the studied mobile pathogens and effectors, the recognition between pathogens and PD, and the antagonistic regulation of PD by plants and pathogens. Finally, we propose several hypothesized strategies to assist hosts in their battle against pathogens via the appropriate modulation of PD.

## Pathogens Exploit Pd to Facilitate Host Infections

**Figure 1:**
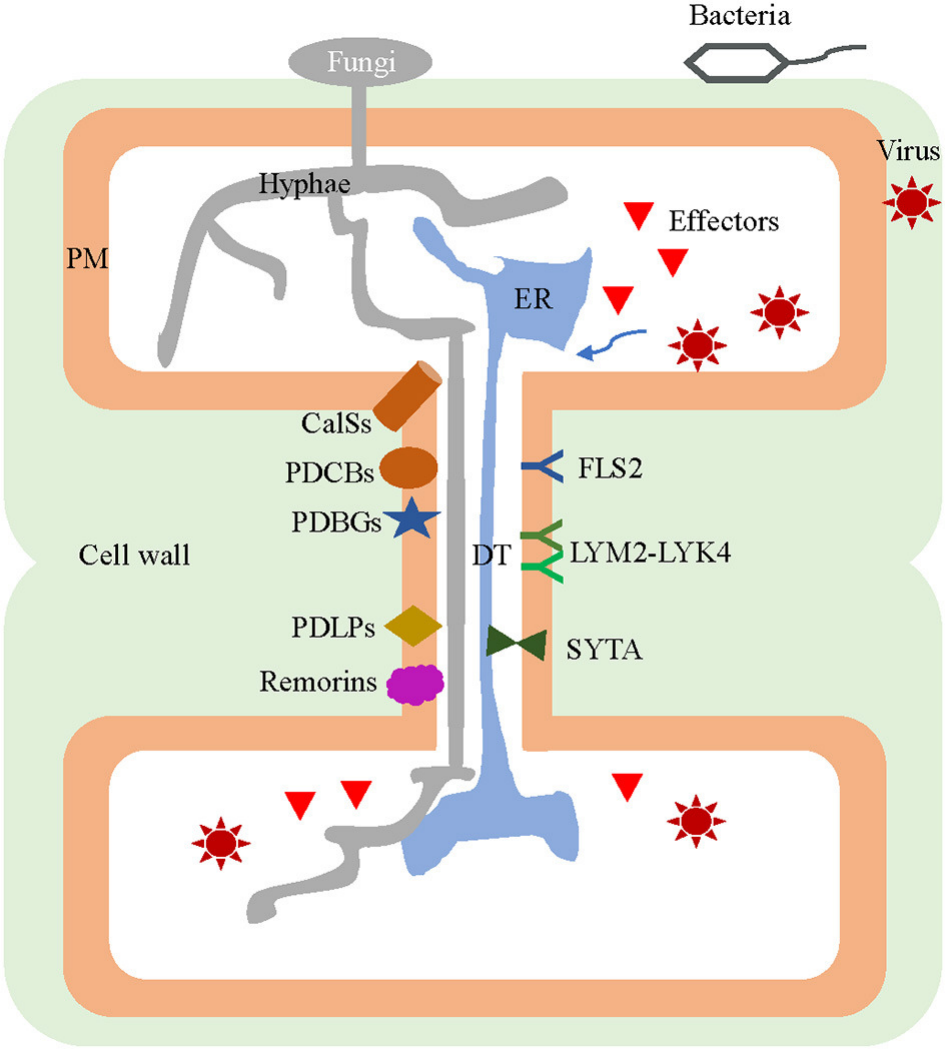
Plasmodesmal components involved in both pathogen infection of plants and host defense responses. Virions, effectors of fungi and bacteria, as well as the fungal hyphae are transported from infected cells to the neighboring healthy cells through the plasmodesmata (PD). Many PD-localized components are jointly exploited by both pathogens and host plants, for use in their interaction during an infection. The pathogens inhibit callose synthesis by inactivating CalSs, suppressing *PDCBs*, destabilizing PDLPs, and/or recruiting PDBGs to assist in their intercellular movement. Viruses can interact with SYTA by relying on MP to remodel the PD. Conversely, PD harbor specific plasmodesmal PM-located receptors of LYM2-LYK4 complex and FLS2 for perception of fungal elicitor chitin and bacterial flagellin, respectively. During infection, some *CalS* and *PDLP* genes can be induced so as to promote callose accumulation and PD closure. The remorins in the membrane microdomains of plasmodesmal PM interact with MP and impede the movement of the virus among host cells. PD, plasmodesmata; DT, desmotubule; CalS, callose synthase; PDCB, PLASMODESMATA CALLOSE-BINDING PROTEIN; PDLP, PD-LOCALIZED PROTEIN; PDBG, plasmodesmal-localized β-1,3 glucanase; SYTA, synaptotagmin A; MP, movement protein; PM, plasma membrane; LYM2, LYSM-CONTAINING GPI-ANCHORED PROTEIN 2; LYK4, LysM-CONTAINING RECEPTOR-LIKE KINASE 4; FLS2, FLAGELLIN SENSING.

Through the PD, the cell-to-cell movement of a variety of molecules is possible. But based on this intercellular connection, phytopathogens have evolved mechanisms that take advantage of PD as gateways to facilitate their host infections (Figure 1). To do this, pathogens encode their own proteins and recruit or interact with the host proteins to target and modify the PD, either directly or indirectly (Table 1).

**Table 1:** Viral, fungal, and bacterial pathogens and the effectors that move cell-to-cell through the plasmodesmata of attacked plants.

	**Pathogens**	**MPs/Effectors**		**Function**	**References**
Virus	Tobacco mosaic virus (TMV)	MP30		MPs bind viral RNAs and increase the SEL of PD in the form of ribonucleoprotein complexes.	Wolf et al., 1989; Brill et al., 2000; Peña and Heinlein, 2012; Pitzalis and Heinlein, 2017
	Carnation mottled carmovirus (CarMV)	P7 and P9			Vilar et al., 2002
	Turnip crinkle virus (TCV)	P8 and P9			Hacker et al., 1992; Li et al., 1998
	Melon necrotic spot virus (MNSV)	P7A and P7B		MPs bind viral RNAs and transit through PD in the form of ribonucleoprotein complexes. TGBp1 of PVX and TGBp2 and TGBp3 of PVX and PMTV increase the PD SEL Tamai and Meshi, 2001; Howard et al., 2004; Haupt et al., 2005	Genoves et al., 2006
	Pelargonium flower break virus (PFBV)	P7 and P12			Martinez-Turino and Hernandez, 2011
	Potato virus X (PVX)	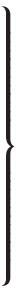	TGB: TGBp1, TGBp2 and TGBp3		Tilsner et al., 2013
	Bamboo mosaic virus (BaMV)				Chou et al., 2013
	Barley stripe mosaic virus (BSMV)				Lim et al., 2008;
	Poasemi latent virus (PSLV)				Shemyakina et al., 2011
	Potato mop-top virus (PMTV)				Zamyatnin et al., 2004
	Beet yellows virus (BYV)	Hsp70h, 64kDa protein, two capsid proteins, and 6-kDa hydrophobic protein			Alzhanova et al., 2000; Dolja, 2003; Avisar et al., 2008
	Tobacco etch virus	Capsid protein (CP)		The CP is required for cell-to-cell and long-distance movement of virus.	Dolja et al., 1995
	Cowpea mosaic virus (CPMV)	MP 58K and 48K	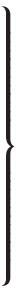	MPs form movement tubles to replace PD desmotubule.	Pouwels et al., 2004; Ritzenthaler and Hofmann, 2007
	Grapevine fanleaf virus (GFLV)	MP 2B			Laporte et al., 2003; Amari et al., 2010
	Cauliflower mosaic virus (CaMV)	MP P1			Thomas and Maule, 1995
	Broad bean wilt virus 2	MP VP37			Xie et al., 2016
	Turnip mosaic virus (TuMV)	6K2 protein		6K2 induces vesicle formation for intercellular movement through PD.	Grangeon et al., 2013
Viroids	Potato spindle tuber viroid (PSTVd)	/		11 RNA loop motifs are critical for cell-to-cell mvement.	Ding et al., 1997; Zhong et al., 2008
Fungi	Magnaporthe oryzae (M. oryzae)	/		IH seek for the pit fields, followed by crossing the PD channels into adjacent cells with constricted hyphae.	Kankanala et al., 2007
		BSA3		BSA3 locates near PD.	Mosquera et al., 2009
		PWL2 and BAS1		PWL2 and BAS1were delivered into the cytoplasm of rice cells by biotrophic interfacial complex (BIC), and finally into neighboring cells via PD.	Khang et al., 2010
	Melampsora larici-populina	AvrL567		AvrL567 accumulates at PD.	Germain et al., 2018
	Ustilago maydis	Cmu1		Cmu1 could likely spread to the neighboring cells through PD and repress SA biosynthesis in host plants.	Djamei et al., 2011
	Phytophthora brassicae	RxLR3		RxLR3 reduce the callose deposition around PD by interacting with and inhibiting CalS1, CalS2, and CalS3	Tomczynska et al., 2020
	Fusarium graminearum	Fusaoctaxin A		Fusaoctaxin A inhibit expression of *PDCB-like* genes and *CalS* genes.	Jia et al., 2019
	Fusarium oxysporum	Avr2 and Six5		Avr2-Six5 effector pair alters plasmodesmatal conductivity to facilitate intercellular movement of Avr2.	Cao et al., 2018
Bacteria	Candidatus Liberibacter asiaticus (CLas)	/		CLas move through sieve pores, and cause callose overproduction and sieve-pore plugging in phloem.	Koh et al., 2012; Achor et al., 2020
	Pseudomonas syringae pv. tomato (Pst) DC3000	HopO1-1		HopO1-1 targets to PD and destabilizes PDLP7 and PLDP5.	Aung et al., 2020
	Pseudomonas syringae pv. tomato (Pst) DC3000	HopK1, HopY1, HopF2, HopU1, HopH1, HopC1, HopN1, HopAA1, HopAF1, HopP1, HopAB2, HopE1, HopAO1, HopA1, HopX1, HopB1		Hop effectors move from infected cells to neighboring cells through PD.	Li et al., 2020

### Viral Spread Through PD

Plant viruses are biotrophic pathogens that utilize the transcriptional machinery of hosts to replicate and propagate with them. To overcome the cell wall barrier, viruses may exploit the PD to engage in cell-to-cell movement and thereby systemically spread throughout the host plants. Viruses encode movement proteins (MPs) to target to and dilate PD (Heinlein and Epel, 2004; Waigmann et al., 2004; Lucas, 2006). For example, a plasmodesmal localization signal sequence in *tobacco mosaic virus* (TMV) and *sugar canemosaic virus* MPs was found necessary and sufficient for PD localization (Yuan et al., 2016; Cheng et al., 2017). Viruses harbor different transport strategies based on differing MP numbers (Epel, 2009). TMV encodes only a single MP, which binds to its RNA and increase the SEL of PD in the form of ribonucleoprotein complexes (Wolf et al., 1989; Brill et al., 2000; Asurmendi et al., 2004; Peña and Heinlein, 2012). In *cowpea mosaic virus, grapevine fanleaf virus*, and *cauliflower mosaic virus*, the MPs reorganize and expand the PD pores by forming a movement tubule (Thomas and Maule, 1995; Laporte et al., 2003; Pouwels et al., 2004). *Carnation mottled virus* encodes two small MPs (DGBp1 and DGBp2) by the double-gene block module (Epel, 2009; Hull, 2014). An early model was proposed, in which DGBp2 interacts with DGBp1:vRNA and drives the transportation of this ternary complex to PD via the endomembrane system. This model is not perfect, however, because of some inconsistencies and the mechanism of *carmovirus* movement is believed to be more complicated (Navarro et al., 2019). The triple gene block module encodes three MPs termed TGBp1, TGBp2, and TGBp3; the TGBp2 and TGBp3 are ER membrane-associated proteins and they form both homologous and heterologous complexes (Morozov and Solovyev, 2003; Lim et al., 2008). Binding of TGBp2/TGBp3 to TGBp1:vRNA allows for the targeting of vRNA to PD and its cell-to-cell spread in the host (Epel, 2009). *Beet yellows virus* (BYV) assembles five MPs to facilitate its intercellular movement, including four viral components-an Hsp70h, a 64kDa protein, and two capsid proteins-and a none-structural 6-kDa hydrophobic protein (Alzhanova et al., 2000). The Hsp70h autonomously targets to PD and its ATPase activity drives the intercellular translocation of BYV (Dolja, 2003; Avisar et al., 2008).

As mentioned before, some plant viruses reorganize the inner structure of PD to produce a movement tubule while passing through it (Thomas and Maule, 1995; Laporte et al., 2003; Pouwels et al., 2004). In this process, MPs are assembled into tubular structures by interacting with the host PDLPs, and this replaces the PD desmotubule to leave only a simple PM-lined tunnel remaining, which aids the viral transport (Amari et al., 2010). The *pdlp1/2/3* triple mutant showed a significant reduction of tubule formation along with diminished local and systemic spread of infection, indicating the important roles of PDLPs (Amari et al., 2010). The cytoskeletons, which are involved in the physical formation and structural operation of PD, are also the modification targets of certain viruses (Liu et al., 2005; Prokhnevsky et al., 2005; Wright et al., 2007; Avisar et al., 2008). One study proved that the MPs of *Cucumber mosaic virus* and TMV are able to sever F-actin, weakening the integrity of PD, thereby allowing larger molecules to pass (Su et al., 2010).

Callose around the PD plays a critical role in regulating their permeability and symplastic communication (Amsbury et al., 2017; Wu et al., 2018). Decreasing this callose was shown to result in an enhanced viral infection (Bucher et al., 2001; Li et al., 2012), whereas increasing callose in the β-1,3-glucanase-deficient and *atbg pap* mutants slowed the spread of the virus (Iglesias and Meins, 2000; Zavaliev et al., 2013). Nevertheless, viruses can facilitate their intercellular movement in hosts by limiting the synthesis of callose and promoting its degradation at PD. For example, *potato virus Y* is capable of inducing the activity of a class I β-1,3-glucanase and suppressing callose accumulation in a strain-nonspecific manner, which may explain why some viruses are still able to spread in resistant-genotype hosts (Chowdhury et al., 2020).

PD is a compelling type of membrane contact site, perhaps best illustrated by the specialization of the ER and the PM at the sites of cell-to-cell junctions (Tilsner et al., 2016). The desmotubule and PM together provide a cytoplasmic conduit for intercellular transport (Roberts and Oparka, 2003; Brunkard et al., 2015). Plant synaptotagmin A (SYTA), a membrane protein, can be recruited to form ER-PM contact sites adjacent to the PD. But viral MPs can interact with SYTA to remodel these contact sites to alter PD and aid viral movement (Lewis and Lazarowitz, 2010; Uchiyama et al., 2014; Levy et al., 2015; Pitzalis and Heinlein, 2017). Recently, the multiple C2 domains and transmembrane region protein family were reported to act as ER-PM tethers specifically at PD (Brault et al., 2019). Further studies need to clearly elucidate the role of the ER-PM membrane in PD functioning and identify more PD tethering machineries that participate in the interactions between pathogens and plants.

Chloroplasts are the organelle responsible for not only the generation of small molecules and secondary metabolites important for plant defense, but also the origination of signals in response to developmental and environmental cues (Ganusova and Burch-Smith, 2019). Nevertheless, particular plant viral proteins can interact with chloroplast proteins to impair the defense of hosts and facilitate the infection of virus (Zhao et al., 2016; Bhattacharyya and Chakraborty, 2018). During Potato virus X (PVX) infection, the viral p25 protein interacts with the chloroplast protein ferredoxin 1 (FD1) to reduce its mRNA and protein levels, resulting in a dramatic decrease of PD callose accumulation that is probably associated with the reduction in phytohormones abscisic acid (ABA) and salicylic acid (SA) (Yang et al., 2020). Arabidopsis *INCREASED SIZE EXCLUSION LIMIT* (*ISE*) *2* encodes a chloroplast DEAH RNA helicase, whose mutation increases the branched PD formation and intercellular trafficking (Kobayashi et al., 2007). The *ISE2* expression can be induced by the infection of TMV or *turnip mosaic virus* in *Nicotiana benthamiana*. However, *ISE2*-overexpressing plants are more susceptible to viral infection, without any influence on callose deposition (Ganusova et al., 2017). These findings imply a still, as of yet unknown mechanism of *ISE2*-mediated chloroplast-nucleus signaling in the interactions between PD and viruses.

Viroids are the smallest known pathogenic agents, consisting only of circular single-stranded RNAs that replicate autonomously and traffic themselves systemically throughout their hosts via the vascular tissue phloem (Flores et al., 2005). Viroids differ from viruses in having unique structural, functional, and evolutionary properties (Flores et al., 2005). Work by Ding et al. (1997) demonstrated that *potato spindle tuber viroid* (PSTVd) can move rapidly from the initially injected mesophyll cells which are interconnected by PD into neighboring cells, whereas it was retained in mature guard cells lacking PD connections. The PSTVd consists of 27 RNA loop motifs flanked by short helices, of which 11 loops were identified as critical for its intercellular movement (Zhong et al., 2008). A small RNA from the virulence-modulating region of PSTVd can suppress the expression of tomato *CalS11-like* and *CalS12-like* genes, pointing to a hypothesized mechanism of viroid movement through PD (Adkar-Purushothama et al., 2015). More mechanisms underpinning the regulation of viroid intercellular trafficking by RNA motifs and cellular factors are reviewed by Takeda and Ding (2009).

### Fungal Infection by Invasive Hyphae (IH) and Effectors

Perhaps the best example of how a fungal pathogen can spread through PD is the study of the hemibiotrophic rice blast fungus, *Magnaporthe oryzae* (M. oryzae; Kankanala et al., 2007; Sakulkoo et al., 2018). By means of the enormous turgor pressure generated by their appressoria, *M. oryzae* breaches the outer cell surface and produces special hyphae named the penetration peg (Howard and Valent, 1996). When entering the epidermal cell lumen, this penetration peg expands to form primary hyphae, which differentiate into bulbous invasive hyphae (IH; Heath et al., 1990). These IH are encased in a plant-derived extra-invasive hyphal membrane outside their cell wall. Then, the bulbous IH seek out pit fields composed of PD clusters in the cell wall, after which they crossing the PD channels into adjacent cells using constricted hyphae (Kankanala et al., 2007). Callose occlusions around the PD were found absent only during the early stages (24–27 h post-inoculation) of invasion in the first rice cell; hence, over this period the PD stayed open, indicating the fungus is able to suppress the callose deposition at pit fields in the host at a specific time before invading the neighboring cells (Sakulkoo et al., 2018). Consistent with this key role of PD, another investigation revealed the failure of IH to move into mature guard cells from neighboring cells due to the degeneration of PD (Kankanala et al., 2007).

Furthermore, the mobile effectors PATHOGENICITY TOWARD WEEPING LOVEGRASS (PWL2) and BIOTROPHY-ASSOCIATED SECRETED (BAS1) produced by *M. oryzae* can move in a cell-to-cell fashion to facilitate host infection (Khang et al., 2010). Both PWL2 and BAS1 are released by IH into the cytoplasm of rice cells by a biotrophic interfacial complex, and move into non-invaded neighboring cells via PD before the spread of IH, which was presumed to better prepare the host cells for the following invasion of IH (Khang et al., 2010). Pmk1, a single fungal mitogen-activated protein kinase, regulates the expression of secreted fungal effectors that inhibit ROS (reactive oxygen species) generation and callose deposition at the PD in rice (Sakulkoo et al., 2018). Accordingly, inhibiting Pmk1 prevents *M. oryzae* from infecting adjacent plant cells, leaving it trapped in the present cell, yet without affecting the biotrophic interfacial complex structure and hyphae morphology (Sakulkoo et al., 2018). These findings indicate the importance of PD for the cell-to-cell invasion of rice cells by *M. oryzae* during the infection process.

The RxLR3 effector produced by *Phytophthora brassicae* can interact with and inhibit CalS1, CalS2, and CalS3, to reduce the callose deposition around PD, so as to promote symplastic trafficking (Tomczynska et al., 2020). In wheat, three *PDCB-like* genes and seven *CalS* genes are suppressed by the virulence factor Fusaoctaxin A during *Fusarium graminearum* infection, which suggests this pathogen may interfere with normal callose accumulation and disrupt the PD status of host plants (Jia et al., 2019). The effectors Avr2 and Six5 secreted by *F. oxysporum* interact at the PD during its infection of tomato; however, Avr2 only moves cell-to-cell in the presence of Six5, while Six5 alone does not alter plasmodesmal conductivity (Cao et al., 2018). Generally, however, the consensus PD-targeting signal peptides of such pathogen effectors have yet to be identified.

### Bacterial Infection With Symplastic Trafficking

Presently, the cell-to-cell spread of bacteria has been mostly reported to occur in the sieve tubes of phloem tissues. *Candidatus* Liberibacter asiaticus (CLas) is a phloem-inhabiting bacterium that causes a destructive disease of citrus trees called Huanglongbing (HLB), which is achieved by its spread via sap flow in the phloem throughout the host plants (Bove, 2006). The cells of CLas adhere to the plasma membrane of those phloem cells positioned specifically adjacent to the sieve pores, and the ensuing morphology changes there enable its movement (Achor et al., 2020). Although we know HLB-infected phloem cells undergo callose accumulation and sieve-pore plugging (Koh et al., 2012; Achor et al., 2020), there is still no evidence showing CLas passing through PD between cells in other plant tissues. The interaction between CLas and phloem cells evidently needs more careful investigation. Usually, bacterial pathogens do not cross the cell wall, probably because their suitable habitat is mostly limited to the apoplastic spaces between plant cells, unlike viruses and fungi which spread intercellularly during local and systemic infections (reviewed by Lee and Lu, 2011). Still, bacteria can release specific effector molecules into plant cells not unlike fungi do, which then move through the PD to spread intercellularly in the host (Li et al., 2020; Figure 1). Only a few effectors have been studied to date. A notable example is the effector protein HopO1-1 of *Pseudomonas syringae* pv. *tomato* (*Pst*) DC3000, a putative mono-ADP-ribosyltransferase. The amino acids in position 41 to 283 (C-terminal end residue) of HopO1-1 are required for its localization to PD (Aung et al., 2020). Once there, HopO1-1 enhances the PD-dependent intercellular molecular flux by destabilizing the PDLP7 and PLDP5 proteins of hosts without affecting their transcript levels (Aung et al., 2020). Further, Li et al. (2020) recently proved that the movement of 16 Hop effectors of *Pst* DC3000 move from transformed cells into neighboring cells through PD depends on their molecular weights. Among them, HopAF1 was characterized by the highest PD-dependent movement, which can nonetheless be inhibited by callose overproduction (Li et al., 2020). This study provided robust evidence that the effectors of bacteria, like fungi, may possess an intercellular mobile ability. It would seem those mobile effectors exploit different mechanisms when interacting with the host during its infection, a topic that warrants further investigation.

## Utilization of Pd by Hosts for Defense

In plant-pathogen interactions, plants have evolved two protein families to recognize pathogens: PM-anchored PRR receptors for PAMPs and intracellular NLR receptors for pathogens effectors (reviewed by Dodds and Rathjen, 2010). The lysin motif (LysM) domain-containing protein CHITIN ELICTOR BINDING PROTEIN (CEBiP) and the receptor-like kinases FLAGELLIN SENSING (FLS2) respectively recognize chitin and flagellin (Kaku et al., 2006; Shimizu et al., 2010; Bücherl et al., 2017). The plasmodesmal PM that is enriched with particular proteins and lipids will integrate extracellular signals differently from the other remaining PM. Increasing numbers of receptors and kinases have been shown to be active in or recruited to plasmodesmal PM (Stahl et al., 2013; Grison et al., 2019; Hunter et al., 2019). A PD-located receptor, LYSM-CONTAINING GPI-ANCHORED PROTEIN 2 (LYM2)/ CEBiP, responds to chitin and signaling, thereby reducing the molecular flux through PD (Faulkner et al., 2013). A receptor complex called LYM2-LYSIN MOTIF-CONTAINING RECEPTOR-LIKE KINASE 4 (LYK 4) (Table 3) found localized at plasmodesmal PM is utilized for plant defense in response to fungal chitin (Cheval et al., 2020). Downstream chitin signaling triggers the phosphorylation of the NADPH oxidase RESPIRATORY BURST OXIDASE HOMOLOG PROTEIN D via a calcium-dependent protein kinase, leading to callose deposition and eventual PD closure. Intriguingly, FLS2 was observed in the vicinity of PD and mediates flg22-triggered changes of PD-mediated trafficking (Faulkner et al., 2013). This phenomenon suggests FLS2 may have an unconsidered role in recognizing flagellin at PD. More receptors at the plasmodesmal PM await discovery.

Being more than simply passive conduits for trafficking, PD also act as hubs capable of integrating multiple signals from the plant development and defense pathways. How do plants protect themselves from pathogens invasion relying on PD? The underlying molecular mechanisms have been elucidated by a few studies. Callose deposition at PD was proven able to restrict infection by pathogen (Cheval and Faulkner, 2018; Wu et al., 2018), suggesting one potential mechanism. The expression levels of *CalS1, 5, 9, 10* and *12* genes were stimulated by *Hyaloperonospora* infection and a SA treatment, whereas the induction of *CalS1* and *CalS12* was significantly repressed in the *npr1* mutant, thus implying a NPR1-dependent regulation (Dong et al., 2008). In the *cals1* mutant, callose at the PD is not affected by either an SA treatment or *Pseudomonas* infection (Cui and Lee, 2016), which suggests CalS1 is essential for SA-mediated callose deposition. The *pdlp1/2/3* triple mutant is more susceptible to the downy mildew pathogen *Hyaloperonospora arabidopsidis*, whereas *PDLP1* overexpression increases callose deposition around the haustoria and enhances plant resistance (Caillaud et al., 2014). PDLP5, localized at the central region of PD, plays a positive role in conferring an enhanced innate immunity of host plants against bacterial pathogens in a SA-dependent manner, by modulating PD callose deposition (Lee et al., 2011; Wang et al., 2013). Enrichment of t18:0-based sphingolipids were found to facilitate the recruitment of PDLP5 proteins to PD, which consequently led to reduced PD conductivity and enhanced resistance to the fungal-wilt pathogen *Verticillium dahlia* and the bacterium *Pst* DC3000 (Liu et al., 2020). Remorins are plant-specific proteins found especially in PM microdomains (Raffaele et al., 2009). Applying SA to plants can trigger a remorin-dependent reorganization of lipid raft nanodomains at PD, thereby modifying the inner structure of PD to impede viral spreading in hosts (Huang et al., 2019). Further, remorins can physically interact with TGBp1, a MP of PVX, to impede the cell-to-cell spread of PVX in tomato leaves (Raffaele et al., 2009).

The number and architecture of PD vary among different cell types and plant developmental stages, which enables the dynamic changes of symplastic transport (Ormenese et al., 2000; Ehlers and Kollmann, 2001; Burch-Smith et al., 2011). During the floral transition of the shoot apical meristem in *Sinapis alba*, for example, the PD frequency increased substantially (Ormenese et al., 2000). While sink leaf cells may contain simple PD in excess of 90%, in stark contrast the source leaf cells mainly contain highly branched PD in *Arabidopsis thaliana*. Correspondingly, the PD in sink cells permit the transport of relatively large molecules, whereas tissues composed of source cells predominantly show a decline in their transport ability (Oparka et al., 1999). *Tomato yellow leaf curl virus* infection leads to an increased number of PD in susceptible tomato plants (Reuveni et al., 2015). Similarly, in *Casuarina glauca* nodules there are fewer PD, perhaps because of the cell enlargement combined with a failed secondary PD formation (Schubert et al., 2013). One study proved ABA negatively regulates PD permeability via callose induction, leading to restricted viral cell-to-cell spreading (Alazem and Lin, 2017). Another study showed treating plants with ABA can modify the number, width, and frequency of their PD (Kitagawa et al., 2019). Collectively, these findings indicate that host plants may reduce and modulate the density and architecture of PD to better defend against invading pathogens. Further investigation is arguably needed to explore in depth the functional PD regulators involved.

## Strategies for Improving Disease Resistance of Hosts By Modulating PD

**Figure 2:**
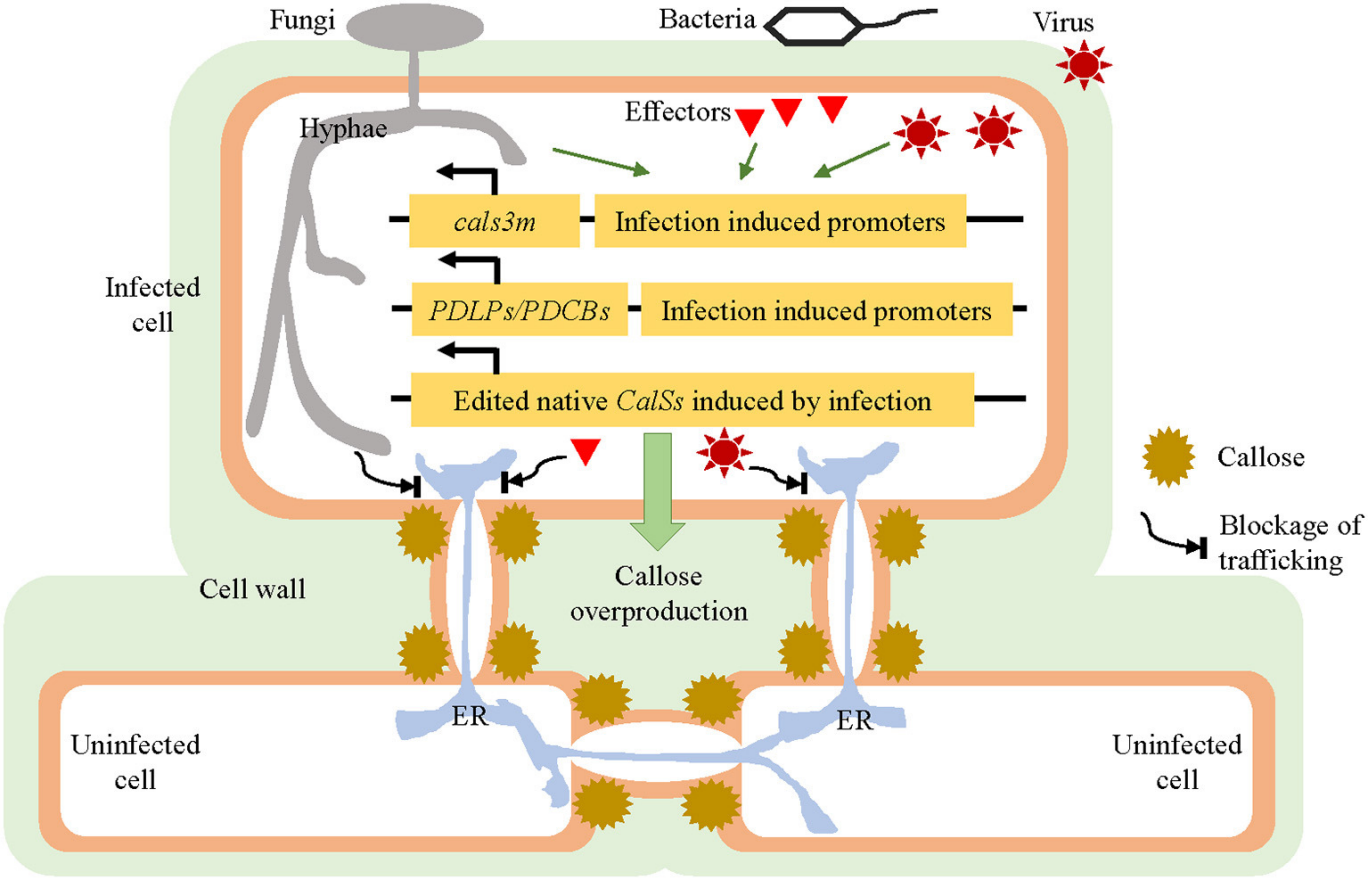
Strategies for improving the disease resistance of hosts by modulating their plasmodesmata (PD). Under normal plant growing conditions, the *cals3m, PDLPs/PDCBs*, and edited *CalSs* genes are expressed at a relatively low level, without affecting the plants' ordinary development. Once a pathogen attack is detected, the expression of *cals3m, PDLPs/PDCBs*, and edited *CalSs* could be induced quickly and strongly, resulting in prompt callose overproduction around the PD and PD closure; this is followed by imposing a blockage of connections between the primary invaded cells and neighboring uninfected cells, which should also stop the intercellular trafficking of pathogens or effectors. These hypothesized approaches are supposed to slow down the spread of pathogens and thus enhance the disease resistance of host plants. PD, plasmodesmata; CalS, callose synthase; PDLP, PD-Localized Protein; PDCB, Plasmodesmata Callose-Binding Protein.

Overall, it is evident that PD can be employed as a weapon, by both pathogens and their hosts, who may compete for control of key PD sites. Although PD confer benefits to both pathogenic infections and their host defense responses (Tables 1, 2), we can try to impede the invasion of one or more pathogens by developing corresponding strategies capable of modifying the PD of the host accordingly. Due to the possible trade-off in functioning between the closure of PD and symplastic transmission of immune signals (Lim et al., 2016b), these strategies must feature quick and effective regulation of PD conductivity spatiotemporally. The prompt and timely induction of PD closure in hosts suffering pathogen attacks are thus speculated to block the trafficking of pathogens, effectors, and toxic molecules from the primary invaded cells into adjacent cells, as well as the needed nutrient import into invaded cells for pathogen growth (Lee et al., 2011; Zavaliev et al., 2011); this might weaken the necessity of systemic immune signal transport. Based on previous findings, we propose three promising hypothesized approaches to spatiotemporally induce callose overproduction and PD closure after pathogen invasion, which would be worth trying to improve plant resistance against enemies (Figure 2).

**Table 2:** Experimentally studied Host proteins/lipids that can regulate the plasmodesmata for plant defense.

**Proteins/lipids**	**Function**	**References**
LYM2-LYK4	PD located LYM2-LYK4 recognize the chitin and trigger downstream signaling to reduce the molecular flux through PD.	Faulkner et al., 2013; Cheval et al., 2020
FLS2	FLS2 is observed in the vicinity of PD and mediates flg22-triggered changes of PD-mediated trafficking.	Faulkner et al., 2013
RBOHD	RBOHD produce ROS that induces PD closure in the signaling cascade of LYM2-LYK4.	Cheval et al., 2020
CalS1	Callose deposition	Dong et al., 2008; Cui and Lee, 2016
CalS12	Callose deposition	Dong et al., 2008
PDLP1	Callose deposition	Caillaud et al., 2014
PDLP5	Callose deposition	Lee et al., 2011; Wang et al., 2013
Calreticulin	Calreticulin interact directly with TMV MP and interferes with targeting of TMV MP to delay cell-to-cell movement of the virus.	Chen et al., 2005
Remorins	Remorins interact with MP TGBp1 of PVX and impairs PVX movement.	Raffaele et al., 2009
	Remorins narrow the PD channels to impede virus spreading depended on SA signaling.	Huang et al., 2019
Sphingolipids	Sphingolipids recruited PDLP5 proteins to PD, which consequently results in the decreased PD conductivity.	Liu et al., 2020

**Table 3:** Abbreviation list.

**Abbreviation**	**Full name**
ABA	Abscisic acid
*A. thaliana*	*Arabidopsis thaliana*
BG	β-1,3 glucanase
PD	Plasmodesmata
CalS	Callose synthase
CEBiP	CHITIN ELICTOR BINDING PROTEIN
DGB	Double gene block
ER	Endoplasmic reticulum
ETI	Effector-triggered immunity
FLS2	FLAGELLIN SENSING
HLB	Huanglongbing
IH	Invasive hyphae
ISE	INCREASED SIZE EXCLUSION LIMIT
LysM	Lysin motif
LYM2	LYSM-CONTAINING GPI-ANCHORED PROTEIN 2
LYK4	LysM-CONTAINING RECEPTOR-LIKE KINASE 4
MAMPs	Microbe-associated molecular patterns
MP	Movement protein
NLR	Nucleotide-binding leucine-rich repeat
PDBG	Plasmodesmal-lacalized β-1,3 glucanase
PDCB	PLASMODESMATA CALLOSE-BINDING PROTEIN
PDLP	PD-LOCALIZED PROTEIN
PM	Plasma membrane
PRRs	Pattern recognition receptors
PSTVd	Potato spindle tuber viroid
PTI	Pattern-triggered immunity
PVX	Potato virus X
SA	Salicylic acid
SAR	Systemic acquired resistance
SEL	Size exclusion limit
SYTA	Synaptotagmin A
TGB	Triple gene block
TMV	Tobacco mosaic virus

### Inducible Callose Overproduction by *icals3m* System

Vatén et al. (2011) developed a system, named *icals3m*, which blocks PD-mediated trafficking by inducing the overproduction of callose surrounding PD in a cell-specific manner. The *icals3m* system has been widely applied to the studies of intercellular trafficking of proteins and small RNAs in biological processes. For example, the symplastic movements of the transcription factor SHORT-ROOT and microRNA165 between the stele and the endodermis were confirmed by the study in plants expressing *pCRE1::icals3m* and *p6xUAS::icals3m* (Vatén et al., 2011). The *cals3m* was also used to investigate cell-cell connectivity between pericycle cells, founder cells, and the neighboring tissues during lateral root formation and patterning in *Arabidopsis thaliana* (Benitez-Alfonso et al., 2013). In the shoot apical meristem, *cals3m* expression could lead to abnormal development and differentiation due to limited movement of WUSCHEL (Daum et al., 2014). Inducible blocking of symplastic signaling going in and out of endodermis by *cals3m* disrupts the coordinated growth and development of roots, which includes an increase of cell layers and the misspecification of stele cells (Wu et al., 2016).

The *icals3m* system provides a wonderful tool for spatially and temporally modulating the aperture of PD. The strategy is to introduce the *icals3m* under the control of pathogen infection-induced promoters into the hosts. Therefore, PD trafficking should get blocked, due to ectopic callose synthesis, once pathogens invade the host cells. The best situation would be that where the attacking pathogens are trapped in primary infected cells without any further spread. In such a case, the usual trafficking of immune signals might not be required even they are also affected. The following three important points likely merit consideration as prerequisites for this approach. First, the promoters must be induced only by pathogen invasion, so they remain inactive or active at very low levels under normal conditions. Otherwise, the callose produced by *cals3m* might interfere with the usual growth and development of host plants. Second, the promoters must respond to the pathogen invasion as soon as possible, preferably prior to the start of its spread into the second plant cell. Third, the induced activities of the promoters must be high enough to yield sufficient callose to constrict the PD. It is known that plant defense responses vary within the same host and among differing ones against different pathogens, so the screening, analysis, and testing for appropriate promoters are crucial steps.

### Inducible Callose Overproduction Utilizing the PDLPs/PDCBs

PDLPs and PDCBs are well known for being positive regulators of callose production. Compared to wild-type plants, overexpression of *PDLP5* restricts the movement of the symplastic tracers CFDA and GFP and some MPs, and conversely the reduction of PDLP5 leads to increased intercellular trafficking (Lee et al., 2011). These findings indicate that changes in *PDLP5* expression were sufficient to regulate both basal PD permeability and MP movement. Similarly, overexpression of *PDLP1* decreased the efficiency of protein diffusion through PD (Thomas et al., 2008). Furthermore, the overexpression of both *PDLP1* and *PDLP5* enhanced plant resistance against pathogens revealing a positive relationship between the levels of PDLPs and plant resistance (Lee et al., 2011; Caillaud et al., 2014). The PDCBs are located at the outer neck region of PD, and greater expression of PDCB1 can lead to increased callose deposition and reduced cell-to-cell trafficking (Simpson et al., 2009). Therefore, we speculate that a timely increase in the expression of PDLPs or PDCBs, or both, could make same contribution to plant defense as *cals3m*. The same selective promoters mentioned above in *icals3m* system may be applied to drive the expression of *PDLPs* and *PDCBs* to increase the callose deposition at the initially infected cells during the onset of infection, thereby preventing pathogens from continuing to invade uninfected tissues. However, a study showed that PDLP5-overexpressing plants are still susceptibility to *turnip crinkle virus* (Lim et al., 2016a), probably due to the ability of virus to alter the aperture of PD (Singh et al., 2017). It is hoped that our approach will prove useful for helping to augment plant resistance to some pathogens to a certain extent. It cannot be expected to inhibit all possible pathogen infections facing host plants due to their different and unknown pathogenic mechanisms.

### Gene Editing of Native Callose Synthases in Hosts

Vatén et al. (2011) identified three allelic semidominant *A. thaliana* mutants called *cals3-1d, -2d*, and *-3d*, which showed aberrant unloading patterns due to the blockage of PD. The *cals3-1d, cals3-2d*, and *cals3-3d* mutations lead to non-synonymous amino acid changes of R84K, R1926K, and P189L, respectively. By combining the two mutations of R84K and R1926K together, the enzymatic activity of encoded callose synthase (cals3m) is increased by 10 to 50% (Vatén et al., 2011). In brief, such mutations in *CALS3* can foster the increased production of callose and reduced aperture of PD that together impair cell-to-cell trafficking activity. This raises an intriguing hypothesis: the introduction of same-site mutations of *cals3m* into other native CalS genes that are quickly and dramatically induced by pathogen attacks, may function similarly as *cals3m*, precluding the introduction of an exogenous gene resource. CRISPR technology is a suitable choice for gene editing (Zaynab et al., 2020). For instance, it was reported that the expression levels of *CalS1* and *CalS12* were highly induced in response to biotic stresses (Dong et al., 2008; Cui and Lee, 2016). Through sequence alignments, we found that both 84R and 1926R of CalS3 are conserved in CalS1 and CalS12 (Supplementary Figure 1), suggesting the feasibility of generating *cals1m* and *cals12m* similarly. However, a pre-test for screening those modifications that do not interfere with the normal functioning of plants in the absence of pathogens is still necessary. When pathogens attack, these improved CalS proteins are then functioning at high efficiency.

## Future Perspectives

Over the last few decades, findings have increasingly emerged which are helpful for addressing how pathogens modify the PD structure and permeability to facilitate their intercellular movement and how plants manipulate PD to impede pathogenic infections. It is known that various PD-localized components are involved in the interactions between pathogens and plants, but many questions about mechanistic differences in how PD are regulated remain unanswered. For example, is there any conserved molecular mechanism conferring symplastic mobility to various pathogens? How do some pathogens or effectors overcome the blockage of PD by callose, and why do others fail to? How do plants manage themselves to gain control over the modification of PD when competing for this with pathogens during an infection? Previously, high-resolution electron microscopy and genetic approaches have greatly advanced our understanding of PD structure and function. Methodological improvements in the isolation and purification of PD may be helpful for identifying new PD components and examining their modifications that occur during interactions between pathogens and plants. PD regulation by pathogens and plants could provide us with a new perspective for the genetic improvement of plant disease resistance.

## Author Contributions

JL, LZ, and DY wrote the manuscript. DY and JL designed the figures. All authors contributed to the article and approved the submitted version.

## Conflict of Interest

The authors declare that the research was conducted in the absence of any commercial or financial relationships that could be construed as a potential conflict of interest.

## Supplementary Material

Supplementary Figure 1 Sequence alignments of callose synthase proteins in *A. thaliana*. Protein sequences from AtCalS1 (NP_563743.2), AtCalS2 (Q9SL03.3), AtCalS3 (Q9LXT9.3), AtCalS4 (Q9LTG5.2), AtCalS5 (Q3B724.1), AtCalS6 (Q9LYS6.2), AtCalS7 (NP_172136.2), AtCalS8 (Q9LUD7.2), AtCalS9 (Q9SFU6.2), AtCalS10 (ACV04899.1), AtCalS11 (Q9S9U0.1), and AtCalS12 (Q9ZT82.1). The red boxes and asterisks denote those amino acids mutated in *cals3m* which are conserved in CalS1 and CalS12.
