# Supplementary material for: Plasmodesmata-Involved Battle Against Pathogens and Potential Strategies for Strengthening Hosts
Source: Front Plant Sci. 2021 Jun 3;12:644870. doi: 10.3389/fpls.2021.644870 (PMC8210831; doi:10.3389/fpls.2021.644870)

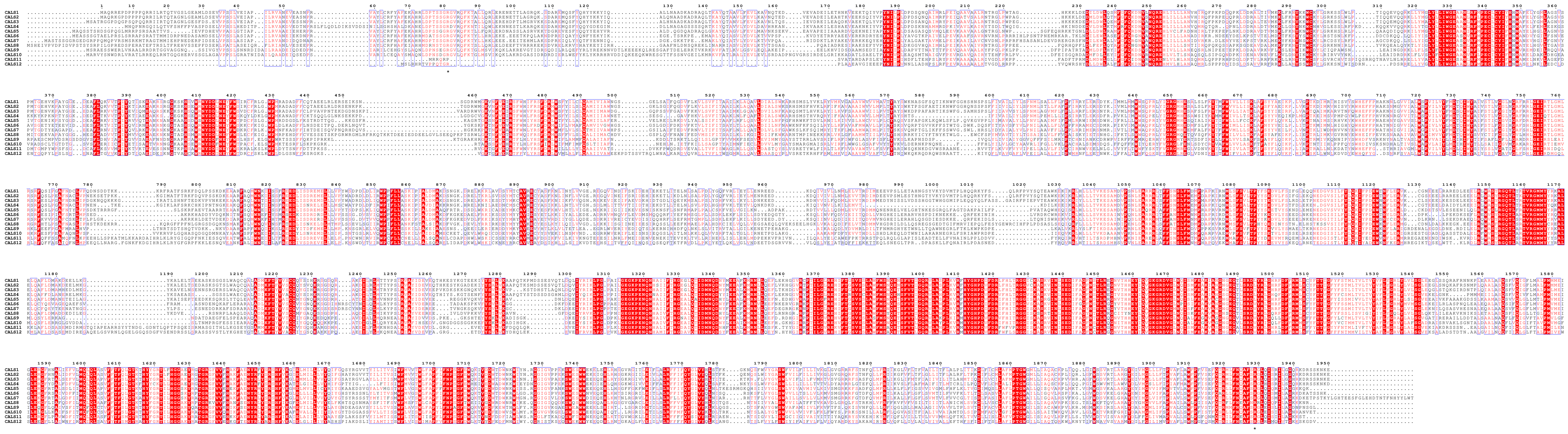

Supplement: Supplementary Figure 1 — Sequence alignments of callose synthase proteins in A. thaliana. Protein sequences from AtCalS1 (NP_563743.2), AtCalS2 (Q9SL03.3), AtCalS3 (Q9LXT9.3), AtCalS4 (Q9LTG5.2), AtCalS5 (Q3B724.1), AtCalS6 (Q9LYS6.2), AtCalS7 (NP_172136.2), AtCalS8 (Q9LUD7.2), AtCalS9 (Q9SFU6.2), AtCalS10 (ACV04899.1), AtCalS11 (Q9S9U0.1), and AtCalS12 (Q9ZT82.1). The red boxes and asterisks denote those amino acids mutated in cals3m which are conserved in CalS1 and CalS12. [file Image_1.pdf]
